# Sericin nanomicelles with enhanced cellular uptake and pH-triggered release of doxorubicin reverse cancer drug resistance

**DOI:** 10.1080/10717544.2018.1469686

**Published:** 2018-05-09

**Authors:** Weihong Guo, Lizhi Deng, Jiang Yu, Zhaoyu Chen, Yanghee Woo, Hao Liu, Tuanjie Li, Tian Lin, Hao Chen, Mingli Zhao, Liming Zhang, Guoxin Li, Yanfeng Hu

**Affiliations:** a Department of General Surgery, Nanfang Hospital, Southern Medical University, Guangzhou, PR China;; b PCFM Lab and GDHPPC Laboratory, School of Materials Science and Engineering, Sun Yat-sen University, Guangzhou, PR China;; c Department of Surgery, City of Hope National Medical Center, Duarte, CA, USA

**Keywords:** Sericin, pH-responsive, micelle, drug resistance, doxorubicin

## Abstract

Drug resistance is the major challenge facing cancer chemotherapy and nanoscale delivery systems based on natural materials, such as sericin, are a promising means of overcoming drug resistance. Yet, no attempt of introducing synthetic poly(γ-benzyl-L-glutamate) (PBLG) onto sericin polypeptide to fabricate a facile biocompatible and biodegradable micelle has been tried. Here, we prepared a polypeptide-based amphiphilic polymer containing hydrophilic sericin polypeptide backbone and PBLG side chains *via* ring-opening polymerization (ROP) strategy. The introduction of PBLG side chains remarkably enhances the stability of sericin micelles in water. Meanwhile, the micelles exhibited a high loading capacity and pH-responsive release ability for antitumor drug doxorubicin (DOX), called sericin-PBLG-DOX. Owing to the excellent cell membrane penetration of sericin-PBLG, the cellular uptake of DOX when loaded into micelles was improved. Subsequently, sericin-PBLG-DOX was transferred into perinuclear lysosomes, where the release rate of DOX was accelerated. Compared to the same dose of DOX, sericin-PBLG-DOX could induce a more efficient anti-tumor effect both *in vitro* and *in vivo,* and these micelles have promise for future clinical applications in overcoming cancer drug resistance with good biosafety, enhanced cellular uptake, pH-triggered drug release, efficient anti-tumor effects, and minimized systemic toxicity.

## 1. Introduction

Fourteen million patients suffered from cancer annually worldwide accounting for over 8 million deaths per year (Siegel et al., 2016). Chemotherapy is the most commonly used cancer treatment for advanced solid malignancies (Kim et al., 2017). Despite the numerous novel chemotherapeutic agents discovered over the years, drug resistance dramatically compromises their effectiveness (Goldman et al., 2016). Chemotherapeutic resistance whether it is inherent or acquired has become the greatest challenge to effective systemic therapy in majority of cancers (He and Shi, 2014). The enhanced activity and high expression of the drug efflux transporter P-glycoprotein (P-gp), which locates on the tumor cell membrane, is critical for drug resistance. The transmembrane structure allows P-gp to actively pump out endocytosed drugs, subsequently reducing the intracellular drug accumulation, and causing reduced chemotherapeutic efficacy (Gupta et al., 2015; Hoosain et al., 2015). High dosage with increased dosing frequency yet yielded clinical benefits, rather often bringing severe adverse side effects to vital organs (heart, liver, and kidney) with a possibility of further worsening drug resistance (Kibria et al., 2014). Development of effective drug delivery systems based on nanotechnology procedures (nano-DDSs) has been proposed as a useful strategy to overcome the drug resistance (Shapira et al., 2011; Markman et al., 2013; Qu et al., 2015).

**Scheme 1.**
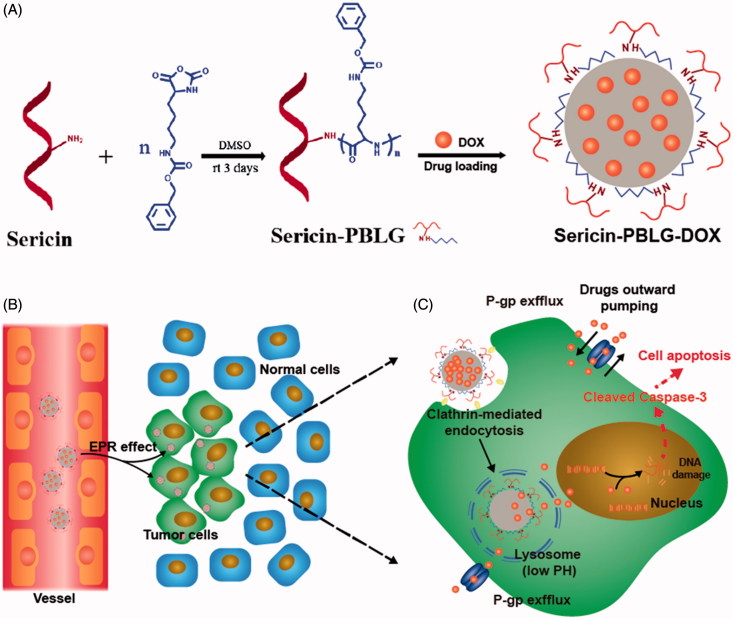
(A) The preparation scheme for sericin-PBLG-DOX micelles. (B) The permeability and retention (EPR) effect of sericin-PBLG-DOX in cancer region. (C) The working routine of sericin-PBLG-DOX during the treatment for drug resistance tumor cells.

The nano-DDSs drugs are considered a promising strategy to reverse drug resistance, for its enhancing intracellular delivery. Meanwhile, previous studies have reported that nanoparticles would be transported into endosomal compartments, such as lysosomes that are in perinuclear regions physically away from the membrane P-gp transporters. This location advantage allows drugs released within these endosomal compartments to reduce the efflux pumping, since the low pH microenvironment of tumor cells and lysosomes could provide a design basis for pH-stimuli responsiveness (Beddoes et al., 2015). *Via* the enhanced permeability and retention (EPR) effect, nano-DDSs can effectively enter tumor region during the blood circulation, compared to the free chemotherapeutic agents (Huwyler et al., 2002; Rajagopal and Simon, 2003; Davis et al., 2008; Yin et al., 2013; Walker et al., 2016; Yang et al., 2016). Under these circumstances, the drugs could directly cross the cell membrane as being warped inside nanocarriers, and released toward the nucleus, thus induced more cancer cells death. Consequently, nanomedicines for enhanced cellular uptake and pH-triggered release of drug, could achieve more efficient anti-tumor effect, since the drug could be taken up and released in such manner.

Regarding the options of materials for nanocarriers, natural polymers have drawn much attention, and have become preferred over recent years. Among all, sericin which is a naturally sticky protein derived from cocoons has drawn great attention. Due to its many advantageous properties, including abundant sources, low immunotoxicity, biodegradability, and abundant modifiable moieties, sericin-based materials are increasingly applied in tissue engineering and biomedicine (Yang et al., 2014; Lamboni et al., 2015). To date, a variety of approaches have been developed to fabricate silk-based nanoparticles, which have been reported in nanocarrier applications for drug and gene delivery, like self-assembled sericin nanoparticles, sericin-PEG nanoparticles, or other kinds of sericin or silk micelles (Vepari and Kaplan, 2007; Mandal and Kundu, 2009; Kundu et al., 2010; Lammel et al., 2010; 2011; Chen et al., 2012; Seib et al., 2013; Xia et al., 2014; Hu et al., 2016; Huang et al., 2016; Liu et al., 2017). Consequently, sericin is regarded as a novel and proper materials in nanocarrier field. Synthetic polypeptides are widely used in biomedical fields, such as drug delivery (Wang et al., 2017) owing to their inherent biodegradable ability and biocompatible degradation products. Among them, synthetic poly(γ-benzyl-L-glutamate) (PBLG) has received much attention, and PBLG has been grafted onto hydrophilic polysaccharide backbone like hyaluronic acid (Upadhyay et al., 2009) or grafting from hydrophilic synthetic polymer such as PEG (Zhang et al., 2016) to form core–shell structured micelles. In such amphiphilic block copolymers, PBLG core serves as reservoir for hydrophobic drugs, greatly increases therapeutic agents’ blood circulation stability. Additionally, PBLG could be degraded into L-glutamic acid (Fang et al., 2015), an important amino acid in human body. The unique advantages make it a good candidate for sericin polypeptide’s modification. To our knowledge, no attempt of introducing PBLG onto sericin polypeptide to fabricate a facile biocompatible and biodegradable micelle has been tried yet.

In this study, we successfully prepared sericin-PBLG derivatives and further synthesized DOX-loaded sericin micelles (sericin-PBLG-DOX) using a dialysis method. We verified the size, zeta potential, drug-loading capability, pH-triggered drug release, and other physical and physicochemical characteristics of these micelles. Then, we constructed DOX-resistant HepG2 hepatoma and MCF-7 breast cancer cell lines, explored the biocompatibility, endocytosis pathway, intracellular localization, controlled drug release, and anti-tumor mechanism of sericin-PBLG-DOX as a drug delivery vehicle during the cancer therapy (Scheme 1).

## 2. Materials and methods

### 2.1. Materials

The silkworm cocoon (*B. mori*) was purchased from Nanning Dongsangxiyi Silk Co. Doxorubicin hydrochloride (DOX·HCl) was obtained from Dalian Meilun (Dalian, China). Dry dimethyl sulfoxide (DMSO), triethylamine (TEA), L-glutamic acid γ-benzyl ester, and triphosgene were obtained from Energy Chemical (Shanghai, China). All other reagents were of analytical grade, and were obtained from Guangzhou Chemical Reagent Co., Ltd. (Guangzhou, China), and used without purification. Male Sprague-Dawley rats, aged 6–8 weeks, were purchased from the Experimental Animal Center of Southern Medical University, which is certified by the Guangdong Provincial Bureau of Science. The rats had been raised in a clean environment, and all animal experiments were performed according to ethical practices. MCF-7 and HepG2 cells were purchased from Cell Bio (Shanghai, China). The cells were cultured in high glucose Dulbecco’s Modified Eagle’s Medium (DMEM; HyClone, Logan, UT) with 10% fetal bovine serum (FBS; Gibco, Brazil), 8 mg/mL penicillin, and 8000 U/mL streptomycin at 37 °C in a humidified 5% CO_2_ incubator.

### 2.2. Synthesis of sericin-PBLG derivatives

#### 2.2.1. Synthesis of BLG-NCA

L-Glutamic acid γ-benzyl ester (5 g) was dispersed in 50 mL of extra dry THF and 3.5 g triphosgene was then added. The reaction was maintained at 55 °C for 1 h under a nitrogen atmosphere, after which the solvent was removed under a vacuum. The oily precipitate was dissolved in 100 mL of ethyl acetate and then washed three times with a saturated NaHCO_3_ solution, and the organic phase collected and dried with anhydrous MgSO_4_. BLG-NCA was obtained after solvent removal under vacuum.

#### 2.2.2. Synthesis of sericin-PBLG

Sericin-PBLG was synthesized by the ring-opening polymerization (ROP) of γ-benzyl-L-glutamic acid-based *N*-carboxyanhydride (BLG-NCA) triggered by the primary amine on the sericin backbone. Typically, 0.5 g of sericin powder, prepared as previously reported (Khire et al., 2010), was thoroughly dissolved in extra dry DMSO at 50 °C under a nitrogen atmosphere and then cooled to room temperature. Following this, 0.2 g of BLG-NCA was added, and the solution was stirred for 3 d at 25 °C. Finally, the product was dialyzed against deionized water for 3 d to remove the organic solvent, and then freeze-dried to obtain the crude product. The powder was then dispersed in 20 mL of DMF by stirring for 30 min and then centrifuged to remove the PBLG homopolymer. Finally, the precipitates were dissolved in DMSO, dialyzed to the remove organic solvent, and freeze-dried to obtain the pure derivative powder sericin-PBLG.

### 2.3. Structural characterization

The element analysis of sericin and sericin-PBLG was conducted on a Vario EL elemental Analyzer (Elementar, Langenselbold, Germany). Each sample was thoroughly mixed with potassium bromide (KBr) and pressed into a pellet form prior to analysis.

### 2.4. Micelle preparation and characterization

The self-assembled sericin-PBLG micelles were prepared *via* a dialysis method. Briefly, 10 mg of sample was dissolved in 1 mL of DMSO. The resulting solution was transferred to a dialysis membrane bag (MWCO = 3500 Da) and then dialyzed against distilled water for 2 d to remove the DMSO. Following this, the micelle solution was adjusted to a total volume of 10 mL, and was then either analyzed or freeze-dried, and stored under refrigeration. The procedure for loading the micelles with DOX differed slightly. First, 2 mg of DOX·HCl was dissolved in DMSO, together with the sericin-PBLG. Then, 40 μL of trimethylamine was added to transform the DOX·HCl into a hydrophobic drug. Subsequently, the organic solvent and any unencapsulated drug were removed by dialysis against distilled water for 24 h. The content of encapsulated DOX was measured at 480 nm using a PerkinElmer UV750 spectrophotometer (PE Co., Waltham, MA). The hydrodynamic diameter and zeta potential of the various micelle samples were measured using a ZetaPALS system (Brookhaven Instruments Corporation, Shanghai, China). The stability of the sericin-PBLG and sericin-PBLG-DOX micelles was measured in phosphate-buffered saline (PBS, pH 7.4, 0.1 M) at 37 °C by measuring their particle sizes at 0.5, 1, 3, 5, and 7 d. Each sample was measured three times independently to obtain an average value. The morphology of the sericin-PBLG and sericin-PBLG-DOX micelles after negative staining with 2% (w/v) phosphotungstic acid solution was imaged with an FEI Tecnai G2 Spirit transmission electron microscope (FEI Co., Eindhoven, Netherlands) operated at 120 kV.

### 2.5. Proton nuclear magnetic resonance (^1 ^H NMR) Fourier transform infrared (FTIR) analyses

Proton nuclear magnetic resonance (^1 ^H NMR) analyses were performed using a Varian INOVA500NB NMR spectrometer (500 MHz; Varian, Inc., Palo Alto, CA, USA) with DMSO-D6 as the solvent. The FTIR spectra were obtained using a Nicolet/Nexus 670 FTIR Analyzer (Thermo Scientific Nicolet, Waltham, MA) at frequencies from 500 to 4000 cm^−1^. Each sample was thoroughly mixed with KBr and pressed into a pellet form prior to analysis.

### 2.6. pH-responsive drug release *in vitro*

Ten Milligrams of lyophilized micelles loaded with 1.5 mg doxorubicin was resuspended in 10 mL deionized water. The samples were transferred into a dialysis membrane bag with a MWCO of 3500, which was immersed in 30 mL buffer at pH 4.5, 6.0, and 7.4, respectively. At predetermined time points, 3 mL of release medium was taken out and 3 mL fresh buffer was added. The content of released doxorubicin was measured by a PerkinElmer UV750 spectrophotometer (PE Co., Waltham, MA) at wavelength of 480 nm.

### 2.7. Hemolysis of sericin-PBLG micelles

Five milliliter of blood samples were collected from healthy individuals in anticoagulant tubes. After collection, the blood was centrifuged at 1500 rpm for 5 min, and then washed twice with PBS. Finally, the red blood cells were dissolved in different final concentrations of sericin-PBLG, while a pure PBS solution (2%, v/v) and Triton X-100 (1%, v/v) were, respectively, applied as negative and positive controls. The mixture was incubated at 37 °C for 4 h, followed by centrifugation at 12,000 rpm for 5 min. Finally, the absorbance (optical density [OD]) of the supernatant was measured at 545 nm and used to analyze the percentage hemolysis.

### 2.8. *In vitro* cytotoxicity assay

MCF-10A cells, LO2 ADR cells, MCF-7 ADR cells, and HepG2 ADR cells were seeded and cultured for 24 h in DMEM with 10% FBS at 37 °C and 5% CO_2_. The cells were then treated with sericin-PBLG, DOX, or sericin-PBLG-DOX solutions at different concentrations.

#### 2.8.1. Cell counting kit 8 (CCK-8) assay

At 48 h, the cells were analyzed using a CCK-8 assay (Dojindo, Kumamoto, Japan) and a microplate reader. The absorbance (OD value) at 450 nm was then determined to assess cell viability. This experiment was repeated 3 times.

To determine whether the intracellular pH-sensitive drug release influence the anti-tumor effect, the seeded cells were co-incubated with NH_4_Cl (20 nM) in the presence of different concentrations of sericin-PBLG-DOX for 48 h. Then, the cell viability was analyzed by CCK8 assay.

#### 2.8.2. Flow cytometry

The cells were washed three times with PBS, trypsinized, and harvested in Eppendorf tubes (Eppendorf, Hamburg, Germany) for staining with an Annexin V-allophycocyanin (APC) apoptosis kit (KeyGEN, Nanjing, China), and 4′,6-diamidino-2-phenylindole (DAPI). The samples were assayed by flow cytometry; APC-positive cells were considered apoptotic cells as determined by the FlowJo software (FlowJo, San Carlos, CA). This experiment was repeated three times.

To further determine whether the clathrin-mediated endocytosis pathways and pH-sensitive drug release influence the anti-tumor effect, the seeded cells were co-incubated with chlorpromazine (10 ug/mL) or NH_4_Cl (20 nM) in the presence of Sericin-PBLG-DOX for 48 h. Then the cell viability was analyzed by Flow cytometry.

#### 2.8.3. 5-Ethynyl-2′-deoxyuridine (EdU) assay

The cells were rinsed three times with PBS and then assessed using the EdU assay (Ruibo, Dongying, China). The cells were observed using a fluorescence microscope.

### 2.9. Cell uptake of sericin-PBLG-DOX

#### 2.9.1. Flow cytometry

MCF-7 ADR cells and HepG2 ADR cells were incubated with sericin-PBLG-DOX (containing 16 ug/mL DOX) for 4 h, harvested, suspended, and analyzed by flow cytometry. The fluorescence channel of DOX was similar to that of PE; consequently, PE-positive cells were considered to have internalized DOX.

#### 2.9.2. Confocal laser scanning microscopy (CLSM) imaging

MCF-7 ADR cells and HepG2 ADR cells were seeded in CLSM dishes, and then treated with a DOX or sericin-PBLG-DOX solution. After incubation for 1–4 h, the cells were washed, fixed in 4% paraformaldehyde, and stained with DAPI. The dishes were imaged by CLSM (Olympus, Tokyo, Japan).

To determine whether tumor cells uptake nanomicelles through clathrin-mediated endocytosis pathways, the seeded cells were pre-incubated with chlorpromazine (10 ug/mL) for 30 min. Then the samples were treated with sericin-PBLG-DOX and further observation by CLSM.

### 2.10. Intracellular distribution of sericin-PBLG-DOX

#### 2.10.1. Transmission electron microscopy (TEM) imaging

MCF-7 ADR cells and HepG2 ADR cells were treated with sericin-PBLG-DOX. After incubation for another 24 h, the cells were harvested and fixed in glutaraldehyde (2.5%), embedded in resin, sliced, stained with osmic acid, and finally viewed using a HITACHI HT7700 system (Hitachi High-Technologies Corporation, Ibaraki, Japan).

#### 2.10.2. CLSM imaging

To further analyze the subcellular localization of sericin-PBLG-DOX after cellular uptake, ADR cells were seeded in CLSM dishes overnight, pre-incubated with 250 nM Lysotracker (KeyGEN, Nanjing, China) for 30 min at 37 °C, and then incubated with DOX or sericin-PBLG-DOX for a further 12 h. Finally, the cells were washed, fixed with 4% paraformaldehyde, stained with DAPI, and imaged by CLSM.

To determine whether the nanomicelles release DOX caused by low pH of lysosome, the seeded cells were pre-incubated with NH_4_Cl (20 nM) for 30 min. Then the cells were treated with sericin-PBLG-DOX and further observation by CLSM.

### 2.11. *In vivo* biosafety study

Sprague-Dawley rats were randomly divided into two groups, the sericin-PBLG and control groups (*n* = 5 per group). After being fasted for one night, these rats were treated with 2 mL of sericin-PBLG or saline *via* tail vein injection. Following this, a blood sample (0.5 mL) was collected from the orbital plexus for liver and kidney function tests. For further biological safety evaluation, the rats were sacrificed using a pentobarbital overdose, and their major organs were harvested for hematoxylin and eosin (H&E) staining.

### 2.12. *In vivo* anti-tumor study

To develop the tumor model, MCF-7 ADR cells and HepG2 ADR cells were harvested and suspended in PBS. Then, 50 μL (at density of 1 × 10^8^/mL) of suspension was injected into the right flank of the mice. Tumor-bearing nude mice were randomly divided into four groups, a saline-treated group as control, a DOX-treated group, a sericin-PBLG-treated group, and a sericin-PBLG-DOX-treated group (*n* = 5 per group). Drugs (5 mg/kg DOX) were administered *via* tail vein injection every 2 d. The tumor volume and body weight were recorded every day for 16 d. After this time, the treated mice were sacrificed and their major organs were excised. The harvested organs were fixed with formalin, followed by immunohistochemistry, and H&E staining.

### 2.13. Western blot analysis

Total protein was extracted from the treated tissue samples. Equal quantities of protein were subjected to the Wes^TM^ full auto western blot assay (ProteinSimple, San Jose, CA). The samples were observed using Compass software (Compass Group, Chertsey, United Kingdom) and a corresponding quantification of the gray value for each protein was determined.

### 2.14. Statistical analysis

Data are expressed as the mean ± standard deviation (SD). All experiments were repeated at least three times with comparable results, unless indicated otherwise. Statistical evaluation of the data was performed using an unpaired Student’s t-test and analysis of variance (ANOVA) followed by Scheffe’s *post-hoc* test. Significant differences were considered when *p* < .05.

Further details about the experimental procedures are included in the supporting files.

## 3. Results and discussion

### 3.1. Physicochemical characteristics

#### 3.1.1. Synthesis of sericin-PBLG-DOX micelles

**Figure 1.**
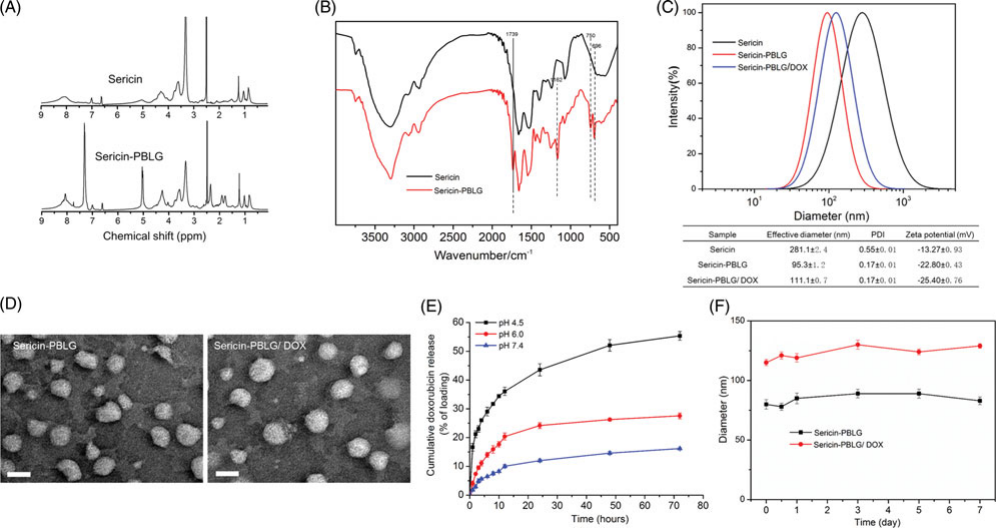
(A) ^1^H NMR spectra of sericin and sericin-PBLG. (B) FTIR spectra of sericin and sericin-PBLG. (C) The particle size and surface charge of sericin, sericin-PBLG, and sericin-PBLG-DOX micelles_._ (D) TEM images of sericin-PBLG and sericin-PBLG-DOX micelles. Scale bar, 50 nm. (E) pH-dependent release of DOX from sericin-PBLG-DOX micelles over 3 d (*n* = 3)_._ (F) Stability of sericin-PBLG and sericin-PBLG-DOX micelles in PBS (pH 7.4, 0.1 M, 37 °C, *n* = 3).

The successful synthesis of sericin-PBLG derivatives is an important step in this approach. To validate the synthesis, we examined the ^1 ^H NMR and FTIR spectra of the sericin and sericin-PBLG derivatives. In the ^1 ^H NMR spectra of the sericin-PBLG derivatives, new ^1 ^H resonance signals appeared at *δ* = 4.95 and *δ* = 7.30 ppm, which could be assigned to the benzene ring protons and benzyl protons of PBLG, respectively (Figure 1(A) (Wang et al., 2017). The grafting of PBLG onto the sericin backbone was further evidenced by the FTIR spectra analysis. As shown in the FTIR spectrum of sericin-PBLG (Figure 1(B)), a strong absorption peak appeared at 1739 cm^−1^, assigned to the stretching vibration of the carbonyl bond (δC=O), at 1162 cm^−1^, assigned to the ester on the benzyl group (δC–O–C) of PBLG, and at 750 and 696 cm^−1^, assigned to the phenyl group in PBLG (Hua et al., 2009; Huang and Jan, 2014). Moreover, we managed to calculate the molecular weight of sericin by sodium dodecyl sulfate polyacrylamide gel electrophoresis (SDS-PAGE) graft ratio of PBLG on the sericin backbone by element analysis, indicating that the molecular weight of sericin ranges from 15 to 70 kDa assay (Figure S1), while the graft ratio was 32.3% (Table S1). Taken together, these data suggest that sericin and PBLG were well conjugated.

#### 3.1.2. Zeta potential and size of the sericin-PBLG-DOX micelles

Figure 1(C) shows that sericin-PBLG micelles prepared by dialysis exhibit a unimodal size distribution. The effective diameter of the sericin-PBLG micelles was ∼100 nm, while DOX loading led to a slight increase in the size of the sericin-PBLG/DOX micelles (110 nm). The introduction of the hydrophobic PBLG side chains onto the sericin backbone helped reduce the particle size of the pure sericin micelles (∼300 nm), which was unsuitable for a drug carrier. In addition, the polydispersity index indicated that the chemical modification led to a more uniform micelle distribution. The data also indicated that loading the micelles with drug led to a slight increase in particle size and a negative net charge, with the overall negative net micelle charge ranging from −15 to −25 mV, while micelles with a negative surface charge have been shown to have improved circulation stability *in vitro* (Alexis et al., 2008).

#### 3.1.3. TEM observation of sericin-PBLG-DOX micelles

As shown in Figure 1(D), the TEM characterization indicated that the sericin-PBLG micelles were spherical in shape, and were approximately 50–100 nm in size, consistent with the dynamic light scattering results. In addition, a slight increase in size was observed after DOX loading. Previous studies have shown that micelles in the sub-100-nm range have enhanced tumor penetration and accumulation (Cabral et al., 2011), indicating that the sericin-PBLG micelles might exert an enhanced anti-tumor effect *in vivo*.

#### 3.1.4. pH-responsive release of DOX from sericin-PBLG-DOX micelles

Increasing the drug concentration in a target region is one main way to improve therapeutic efficacy. As such, achieving the release of a medicine in a certain region is important. Typically, the pH microenvironment of tumors and lysosomes is much lower than that of normal tissues. As such, micelles with a low pH-triggered DOX release mechanism that allows for accelerated drug release in cancer containing regions show promise for clinical application as a nanomedicine.

The drug loading efficiency (LE) was 13.8%, which was determined as follows: LE = number of DOX equivalents in the micelle/amount of polymer. To evaluate the release curve of sericin-PBLG-DOX micelles, the *in vitro* release of DOX was monitored in buffers with pH values mimicking those of blood plasma (pH 7.4), early endosomes (pH 6.0), and lysosomes (pH 4.5) (Hu et al., 2016; Huang et al., 2016). As shown in Figure 1(E), throughout a 72-h release period, the release rate of the drug was significantly accelerated at a mildly acidic pH (4.5 and 6.0). In particular, when incubated at pH 4.5, approximately 50% of the DOX was released into the medium over 72 h. In order to achieve this low pH-dependent release, doxorubicin hydrochloride, a soluble positively charged drug, was neutralized with trimethylamine to produce hydrophobic doxorubicin (DOX) which could be readily encapsulated into the hydrophobic core of the sericin-PBLG micelles through hydrophobic interactions. And sericin is abundant of side carboxyl groups, which contains 18% aspartic acid (Cao and Zhang, 2016). The DOX-loaded micelles were constructed in a neutral environment, leading to an acidic surface with negative net charge, which helps to tightly encapsulate the hydrophobic alkaline DOX. At a lower pH, the carboxyl groups from aspartic acids become protonated, and the electrostatic repulsion between the sericin-PBLG micelles is consequently weakened, resulting in swelling of the micelles, and leading to an acceleration in the rate of DOX release from the sericin-PBLG micelles. A similar pH-dependent release behavior for DOX has also been observed in nanoparticles fabricated using silk protein (Seib et al., 2013). Consequently, we expected to achieve an improved anti-tumor effect since the drug could be released in a sustained manner.

#### 3.1.5. Sericin-PBLG-DOX micelle stability

Finally, we investigated the stability of the sericin-PBLG and sericin-PBLG-DOX micelles in PBS (pH 7.4, 0.1 M) at 37 °C, as shown in Figure 1(F). The sericin-PBLG micelles remained stable over a week, while DOX was encapsulated without precipitation.

### 3.2. *In vitro* and *in vivo* biocompatibility of sericin-PBLG micelles

**Figure 2.**
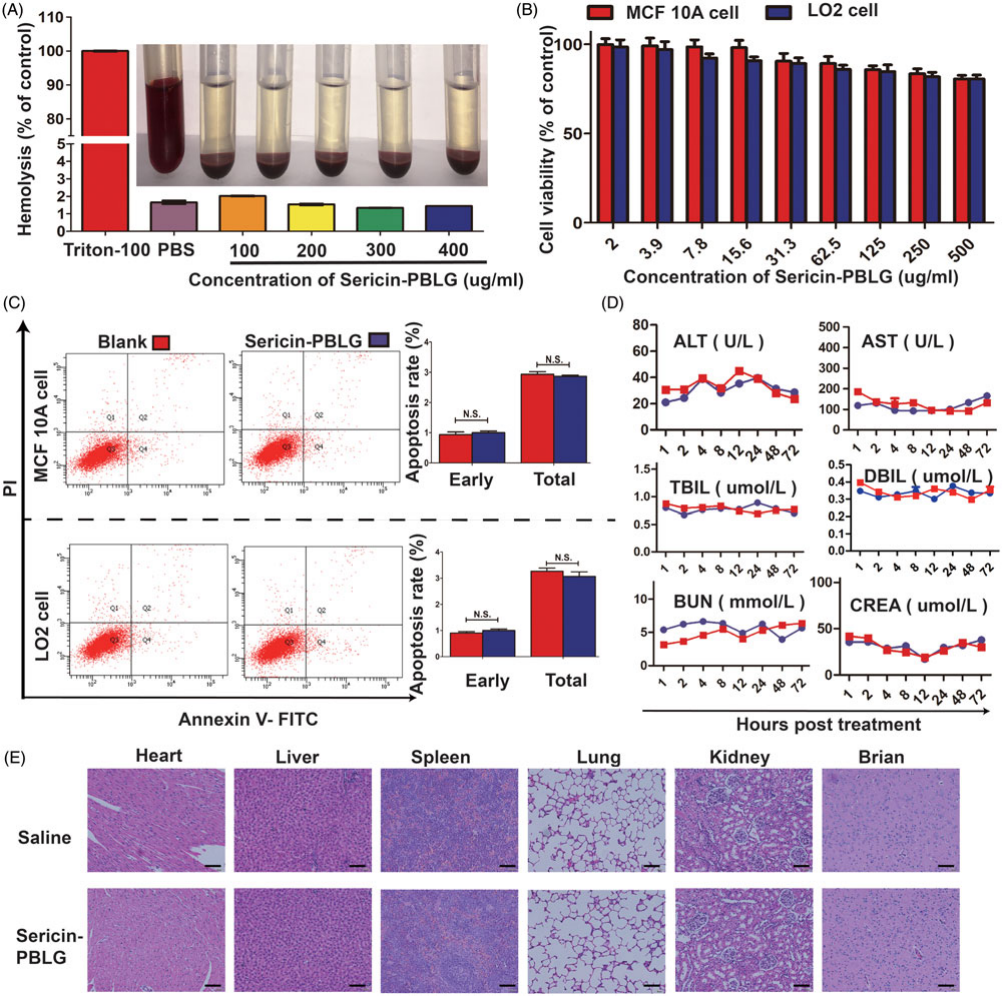
*In vitro* and *in vivo* biocompatibility of sericin-PBLG. (A) Assessment of the potential for hemolysis by different concentrations of sericin-PBLG nanomicelles. Triton X-100 was used as a positive control, and PBS was used as a negative control. (B) Cell viability of MCF-10 A cells and LO2 cells after incubation with sericin-PBLG. (C) Apoptosis rates of MCF-10 A cells and LO2 cells after incubation with sericin-PBLG. Data are shown as the mean ± SD, *n* = 3, N.S. indicates no significant difference. (D) Assessment of biochemical parameters in sera collected from mice treated with PBS or sericin-PBLG. AST, aspartate aminotransferase; ALT: alanine aminotransferase; TBIL: total bilirubin; UBIL: unconjugated bilirubin; BUN: blood urea nitrogen; UA: blood uric acid. (E) Histopathological analysis of major organ tissues isolated from Sprague-Dawley rats. Scale bar, 50 nm. Data are shown as the mean ± SD, *n* = 5 animals per treatment.

*In vitro* experiments were used to address biocompatibility by incubating the sericin-PBLG micelles with red blood cells. The sericin-PBLG micelles did not induce hemolysis at any concentration tested (ranging from 100 to 400 µg/mL), as shown in Figure 2(A). A CCK-8 assay (Figure 2(B)) indicated that there was only minor toxicity toward two types of normal breast and liver cells (MCF10A and LO2), as the cell viability rates were all above 75% after 48 h of incubation with different concentrations of sericin-PBLG ranging from 2.0 to 500 µg/mL. Consistent with this high biocompatibility, cells treated with sericin-PBLG at a concentration of 250 μg/mL, showed no significant difference in the early or total apoptosis rates (Figure 2(C)). The immunotoxicity of sericin-PBLG was also examined using murine macrophage-like cells (RAW264.7), indicating that sericin-PBLG treatment did not elicit inflammatory responses at the cellular level (supporting materials, Figure S2). Taken together, these data strongly suggest that sericin-PBLG micelles have good *in vitro* biocompatibility (Hekmat et al., 2016; Jin et al., 2016).

To further evaluate the potential toxicity of this new nanoscale drug delivery system, we next assessed the systemic biosafety of sericin-PBLG *in vivo*. Sprague-Dawley rats received sericin-PBLG (10 mg/kg) or saline treatment *via* tail vein injection (*n* = 5). Following this, blood serum samples were sent for biochemical analysis to assess clinically relevant indicators, aspartate aminotransferase, alanine aminotransferase, total bilirubin and unconjugated bilirubin for the liver, and creatinine, and uric acid for the kidneys. No significant differences were observed between the sericin-PBLG and saline control groups, indicating that sericin-PBLG did not induce damage to the liver or kidneys (Figure 2(D)). Moreover, a histological analysis of the main organs (heart, liver, spleen, lungs, kidneys, and brain) using H&E staining further confirmed the *in vivo* biosafety of sericin-PBLG, as there were no pathological changes compared with the control group (Figure 2(E)). Taken together, these data provide strong evidence that sericin-PBLG micelles induce negligible systemic toxicity, suggesting their potential value for clinical use as a drug carrier.

### 3.3. Sericin coating promotes DOX cellular uptake through the clathrin-mediated endocytosis pathway

As described above, the biosafety of the sericin-PBLG nanocarrier was validated both *in vitro* and *in vivo*, and the high biocompatibility observed demonstrates the further potential for use as in cancer treatment. However, the drug delivery mechanism for sericin-PBLG-DOX is unknown, which is a critical aspect of pharmaceutical research (Andre et al., 2016). Nanoscale drugs are considered to be a promising strategy for enhancing the intracellular delivery of drugs. One of the advantages of nanoparticles is that they can enhance the water solubility of hydrophobic drugs, which could be easily achieved through packaging with an amphiphilic carrier (XuShi et al., 2015). In such cases, the cancer cells can be more easily killed since more drugs can be taken up.

**Figure 3.**
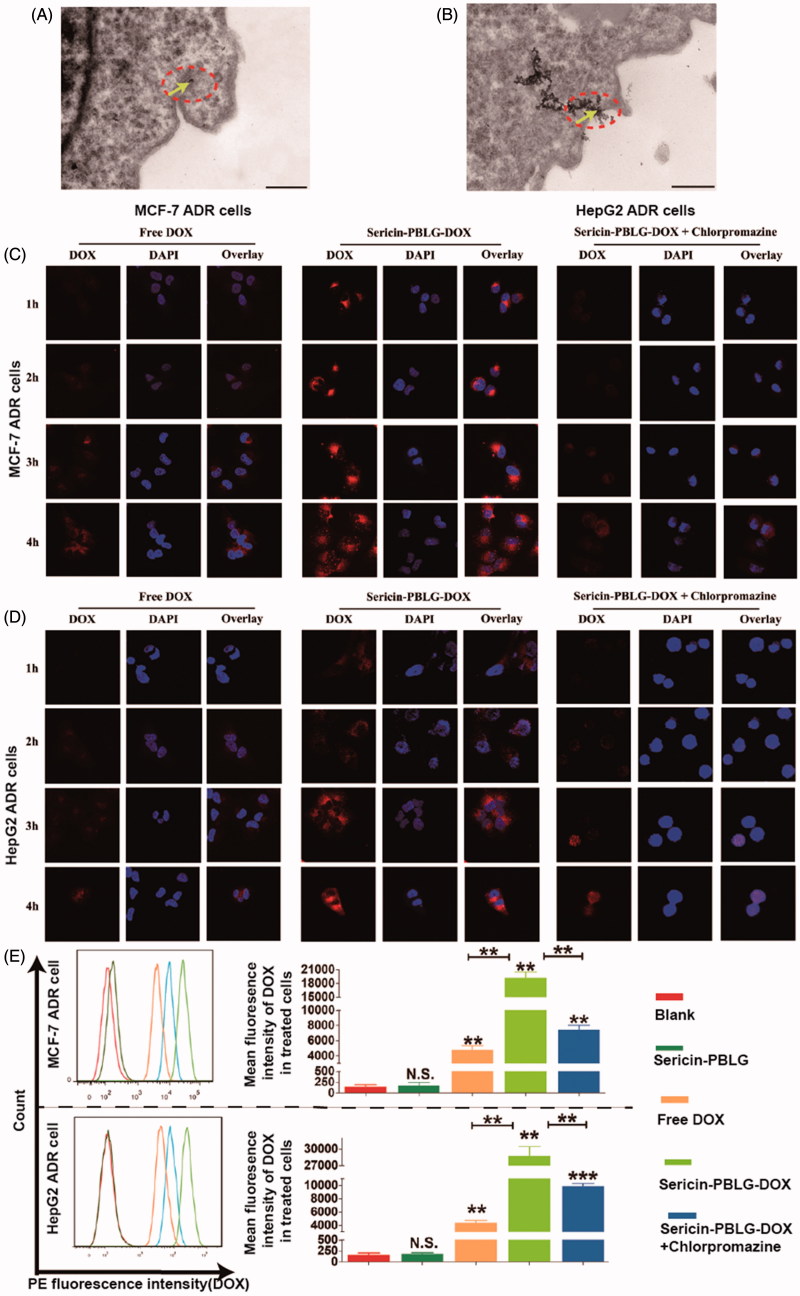
Cell uptake mechanism of sericin-PBLG-DOX. TEM images of the endocytosis of sericin-PBLG-DOX nanoparticle by MCF-7 ADR cells (A) and HepG2 ADR cells (B), the yellow arrows represent nanomicelles. Scale bar, 500 nm. CLSM images of MCF-7 ADR cells (C) and HepG2 ADR cells (D) treated with DOX, sericin-PBLG-DOX, or sericin-PBLG-DOX plus chlorpromazine. Scale bar, 500 nm. (E) The representative images (left) of the flow cytometry analyses of MCF-7 ADR and HepG2 ADR cells treated with sericin-PBLG, free-DOX, sericin-PBLG-DOX, and sericin-PBLG-DOX plus chlorpromazine for 4 h (left) and the corresponding quantification of the mean DOX red fluorescence intensity (right). Data are shown as the mean ± SD, *n* = 3. N.S. indicates no significant difference, ** indicates *p <* .01 and *** indicates *p <* .001.

To examine the uptake mechanism more closely, we first observed the cellular uptake of the sericin-PBLG-DOX micelles. TEM images (Figure 3(A,B)) showed that a number of black particles could be observed around the cell membrane. In addition, dozens of vesicles containing black particles were also observed inside the cell cytoplasm (supporting materials, Figure S3), which suggested that the black particles might be sericin-PBLG-DOX micelles (Zhang et al., 2012; XuShi et al., 2015). Owing to the natural ability of sericin to adhere to the cell surface (Wang et al., 2014), we hypothesized that the sericin-PBLG coating mostly increased the cellular uptake of DOX by the clathrin-mediated pathway, which is a classic endocytosis uptake mechanism (Merrifield and Kaksonen, 2014). However, it was hard to validate that the round objects observed were indeed micelles, and thus the mechanism of cellular delivery was not completely proven.

In order to examine the uptake mechanism further, DOX-resistant cells, including MCF-7 ADR and HepG2 ADR cells, were created (supporting materials, Figure S4). We compared the cellular uptake efficiency of sericin-PBLG-DOX at a concentration of 16 µg/mL with that of free DOX in MCF-7 ADR cells through CLSM. The CLSM images (Figure 3(C)) suggested that the absorption of DOX increased gradually, reaching its peak at 4 h. Compared to the free DOX-treated cells, more DOX could be absorbed by sericin-PBLG-DOX-treated cells over 1 and 4 h, suggesting that sericin-PBLG significantly increases the uptake of DOX into MCF-7 ADR cells. To further confirm the endocytosis pathway, we pre-incubated the ADR cells with chlorpromazine (10 μg/mL), a specific clathrin protein inhibitor (Mayor and Pagano, 2007). The uptake of sericin-PBLG-DOX was significantly suppressed in MCF-7 ADR cells, implying that sericin-PBLG enhances the uptake of DOX through the clathrin-mediated endocytosis pathway. HepG2 ADR cells showed similar results (Figure 3(D)). In addition, the cellular uptake of DOX in cancer cells was also validated by flow cytometry. Since the fluorescence channel for DOX is similar to PE, PE-positive cells were considered to have internalized DOX in this study. A quantitative analysis obtained from the flow cytometry study (Figure 3(E)) indicated that almost no fluorescence was observed in both the blank and sericin-PBLG-treated cells, whereas a marked increase in fluorescence was observed in the free DOX, sericin-PBLG-DOX, and sericin-PBLG-DOX plus chlorpromazine-treated cells. Furthermore, the mean fluorescence intensity in the sericin-PBLG-DOX-treated cells was significantly increased, compared to the free DOX-treated cells. Again, the fluorescence signal was dramatically suppressed in cells also treated with chlorpromazine. The corresponding mean PE fluorescence intensity remained largely consistent with the CLSM results. Collectively, these data demonstrated the important functional role of sericin-PBLG micelles in promoting the cellular uptake of DOX through the clathrin-mediated endocytosis pathway. However, these experiments only provided information on the relative DOX content in cancer cells, and in the future a more exact method may be required to analyze the DOX levels inside the ADR cells, such as high-performance liquid chromatography (HPLC) or liquid chromatography-mass (LCMS).

### 3.4. pH responsiveness ensures effective DOX release from sericin-PBLG-DOX toward the nucleus

**Figure 4.**
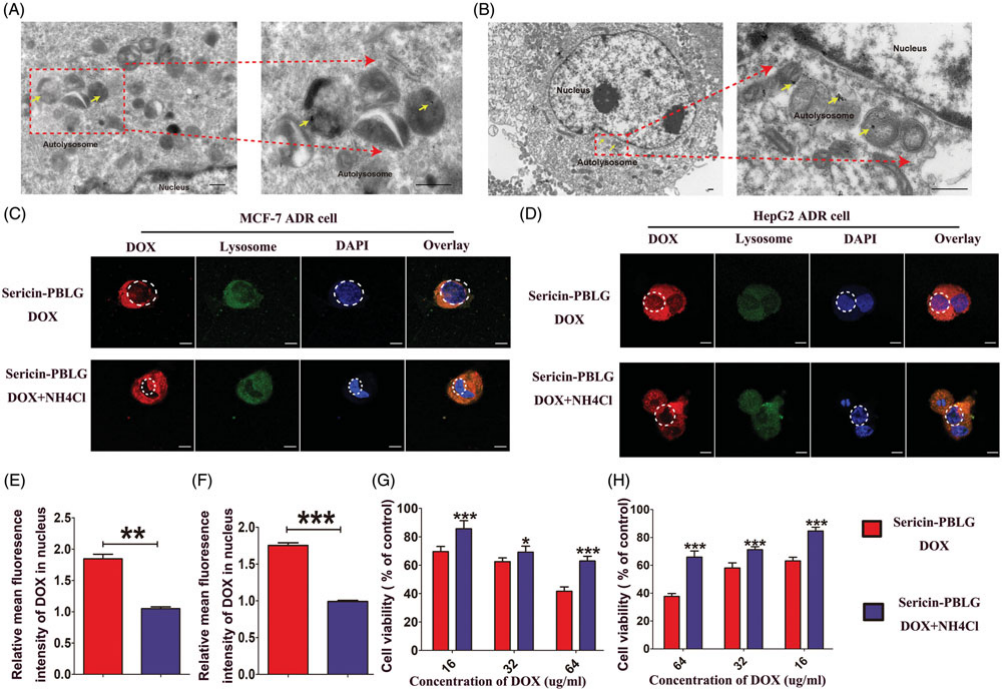
Intracellular distribution and pH-triggered drug release of sericin-PBLG-DOX. TEM images of MCF-7 ADR cells (A) and HepG2 ADR cells (B) after incubation with sericin-PBLG-DOX. Scale bar, 500 nm. CLSM images of MCF-7 ADR cells (C) and HepG2 ADR cells (D) with or without NH_4_Cl treatment in the presence of sericin-PBLG-DOX nanoparticles. Scale bar, 100 nm. The relative mean fluorescence intensity of DOX in the nucleus of MCF-7 ADR cells (E) and HepG2 ADR cells (F). Viability of MCF-7 ADR cells (G) and HepG2 ADR cells (H) with or without NH_4_Cl treatment in the presence of sericin-PBLG-DOX nanoparticles. The yellow arrows represent nanomicelles. Data are shown as the mean ± SD, *n* = 3. * indicates *p <* .05, ** indicates *p <* .01, and *** indicates *p <* .001.

Although we had demonstrated the cellular uptake mechanism, the intracellular processes remained unclear. During cellular uptake, nano-DDSs are thought to be absorbed by tumor cells, and then transported into the endosomal compartment, such as lysosomes that lie in the perinuclear region. This location provides the advantage of allowing drugs to be released from these endosomal compartments and then being delivered to the nucleus (Beddoes et al., 2015). To clarify the intracellular processes used by sericin-PBLG-DOX, we first observed the intracellular distribution by TEM. After incubation with MCF-7 ADR cells and HepG2 ADR cells for 24 h, autolysosomes were observed around the nucleus, the latter containing dozens of black particles, which were thought to be the sericin-PBLG-DOX micelles (Figure 4(A,B)).

This phenomenon suggests that sericin-PBLG-DOX might have entered the lysosomes (Zhang et al., 2012; XuShi et al., 2015). To further address this issue, MCF-7 ADR cells were first labeled with a lysotracker probe, which exhibits green fluorescence when taken up into lysosomes. From the CLSM images (Figure 4(C)), the location of sericin-PBLG-DOX largely overlapped with that of the lysosomes. As shown previously (Figure 1(F)), the sericin-PBLG-DOX micelles exhibited an accelerated release of DOX due to the low pH-triggered release mechanism. Lysosomes, which are a degradative organelle, have a much lower pH microenvironment inside tumor cells (Lee et al., 2015; Wang et al., 2016). Consequently, we hypothesized that after cellular internalization, sericin-PBLG-DOX which localizes in the lysosomes might show an accelerated release of DOX, with the result that the DOX will be directed toward the nucleus due to the special pH microenvironment, thus effectively exerting an anti-tumor effect (Seib et al., 2013; Wang et al., 2015).

In order to verify this further, we pre-incubated MCF-7 ADR cells with NH_4_Cl, a specific inhibitor that neutralizes acidic microenvironments (Jansen et al., 2008). Compared with the sericin-PBLG-DOX-treated cells, the nuclear accumulation of DOX was significantly reduced in the NH_4_Cl-treated cells (Figure 4(C,D)). Consistent with this, the relative mean fluorescence intensity of DOX in nucleus (Figure 4(E,F)) showed that the nuclear DOX levels were dramatically reduced in the presence of NH_4_Cl. These data further demonstrate the low pH-dependent triggering of DOX release from the sericin-PBLG-DOX micelles. However, whether this then improves therapeutic efficacy, remained unknown. Accordingly, we further compared the anti-tumor effect of sericin-PBLG-DOX in the presence or absence of NH_4_Cl. Cell viability was increased in the NH_4_Cl-treated cells (Figure 4(G,H)), indicating that the acidic inhibitor NH_4_Cl could effectively reduce DOX release, leading to a less effective anti-tumor effect.

Taken together, these results demonstrate that the nanocarrier sericin-PBLG-DOX can efficiently promote the cellular uptake of DOX, and enhance DOX accumulation in the nucleus through a pH-responsive release mechanism.

### 3.5. Sericin-PBLG-DOX reverses the DOX-resistance of cancer cells *In vitro*

Because of their ability to enhance intracellular delivery, their responsive release mechanism, and their more efficient anti-tumor effect, nanoscale drugs are considered to be a promising strategy to reverse drug resistance. Previously, we have demonstrated the cellular uptake of DOX through the clathrin-mediated endocytosis pathway, and its pH-triggered release. Based on these data, we hypothesized that sericin-PBLG-DOX might be able to reverse resistance of cancer cells to DOX, since the sericin coating promotes DOX cellular uptake.

**Figure 5.**
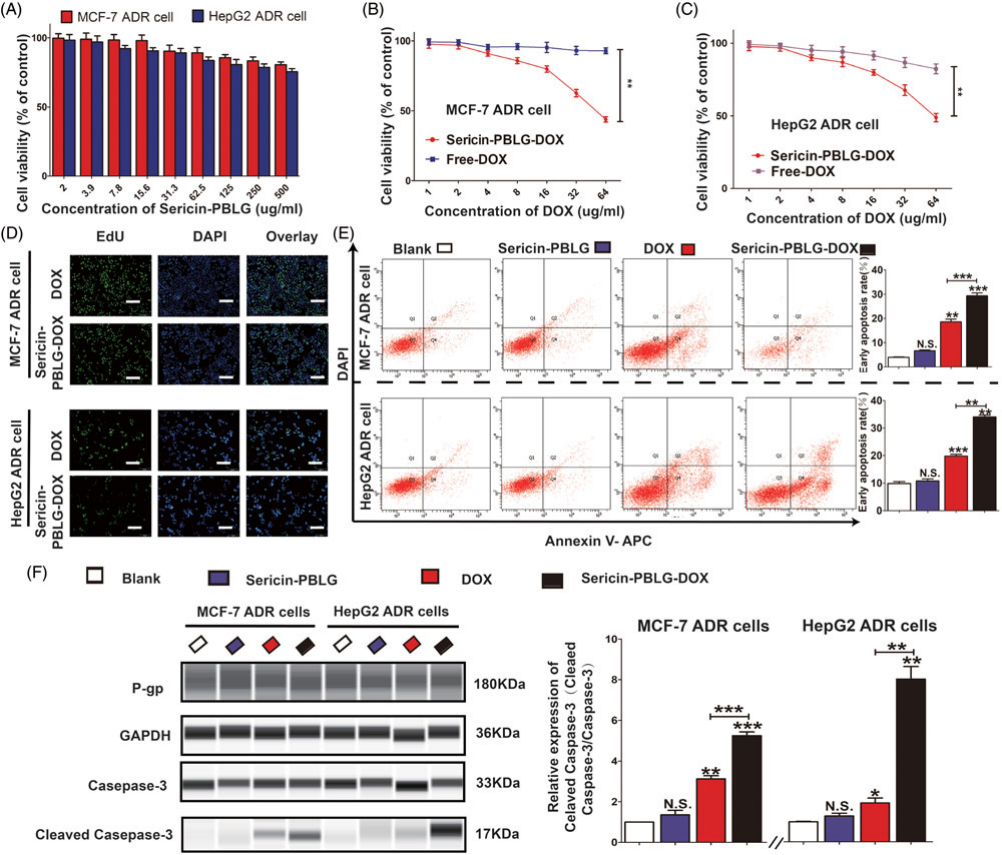
*In vitro* anti-tumor effect of sericin-PBLG-DOX. (A) Viabilities of MCF-7 ADR cells and HepG2 ADR cells after incubation with the sericin-PBLG nanocarrier. (B and C) Viabilities of MCF-7 ADR cells and HepG2 ADR cells after incubation with DOX or sericin-PBLG-DOX. (D) EdU assay of MCF-7 ADR cells and HepG2 ADR cells after incubation with DOX or sericin-PBLG-DOX. Scale bar, 200 nm. (E) Apoptosis rates of MCF-7 ADR cells and HepG2 ADR cells after incubation with DOX or sericin-PBLG-DOX. (F) Protein expression of P-pg, caspase-3, cleaved caspase-3 protein, and GAPDH in MCF-7 ADR cells, and HepG2 ADR cells after incubation with sericin-PBLG, DOX, or sericin-PBLG-DOX for 48 h (left), and the corresponding quantification of the gray value for each protein (right). Data are shown as the mean ± SD, *n* = 3, N.S. indicates *p >* .05, * indicates *p <* .05, ** indicates *p <* .01, and *** indicates *p <* .001.

The anti-tumor effect was assessed by incubating sericin-PBLG micelles with MCF-7 ADR and HepG2 ADR cells. Sericin-PBLG micelles were relatively nontoxic to ADR cells, as the cell viability rates were all above 75% after 48 h of incubation with different concentrations of sericin-PBLG ranging from 2.0 to 500 µg/mL (Figure 5(A)). In parallel, incubating cells with increasing concentrations of DOX for 48 h showed a significant dose-dependent decrease in cell viability as measured by CCK-8 assay, with sericin-PBLG-DOX inducing a much greater anti-tumor effect in ADR cells than free DOX (Figure 5(B,C)). To further examine the anti-tumor effect, we incubated the ADR cells with sericin-PBLG-DOX or free DOX at a concentration of 16 µg/mL for 48 h. An EdU incorporation assay (Figure 5(D)) revealed that there were less EdU-positive cells in the sericin-PBLG-DOX-treated cells than in the free DOX-treated cells. Consistent with this data, when MCF-7 ADR cells were analyzed by flow cytometry (Figure 5(E)) sericin-PBLG-DOX was found to induce significantly more apoptosis (1.5-fold higher) in MCF-7 ADR cells (30%) than free DOX (18%). HepG2 ADR cells were also analyzed by flow cytometry (Figure 5(E)) showing that sericin-PBLG-DOX-induced significantly more apoptosis (1.5-fold higher) (34%) than free DOX (19%). Our data also demonstrated that the fluorescence of the DNA damage sensor γH2AX (Lv et al., 2018) was also dramatically elevated (supporting materials, Figure S5) in sericin-PBLG-DOX-treated cells. Consistent with this FACS data, the expression levels of well-defined apoptosis protein markers (cleaved caspase-3/caspase-3) (Chang et al., 2016; Rashidi et al., 2018), were found to be markedly increased in sericin-PBLG-DOX-treated cells, compared to the free DOX-treated cells (Figure 5(F)). Although the expression levels of the apoptosis marker proteins in the free DOX-treated cells were up-regulated, the levels were still significantly lower than the levels seen in the sericin-PBLG-DOX-treated cells. In contrast, sericin PBLG itself was not seen to cause changes in cell proliferation or apoptosis, compared to control cells. These results indicate that sericin-PBLG-DOX can efficiently inhibit tumor cell proliferation, and this enhanced effect was mainly due to sericin-PBLG-DOX, and not to sericin-PBLG itself.

The enhanced activity and high expression of the drug efflux transporter P-gp, which is located on the tumor cell membrane, is critical for drug resistance (Gupta et al., 2015; Dewanjee et al., 2017). In our drug-resistant ADR cells, the expression of P-gp was much higher than in drug-sensitive cells (supporting materials, Figure S6). However, the expression of P-gp was unchanged in the sericin-PBLG-DOX-treated cells (supporting materials, Figure S7), indicating that sericin-PBLG-DOX did not reduce the expression of P-gp.

These results indicate that sericin-PBLG-DOX, but not free DOX or sericin-PBLG, can effectively eliminate drug-resistant cancer cells by enhancing the intracellular delivery of drug, instead of inhibiting P-gp expression. Further research is still needed to explore the mechanism of action in more detail.

### 3.6. Intravenous sericin-PBLG-DOX administration effectively inhibits DOX-resistant tumor growth without eliciting systemic toxic side effects

**Figure 6.**
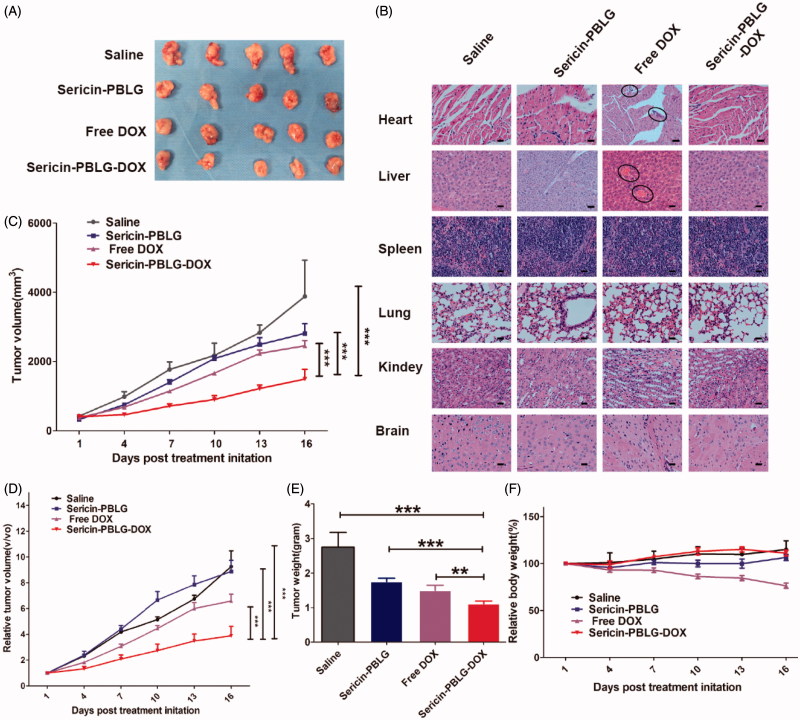
*In vivo* anti-tumor effect of sericin-PBLG-DOX. (A) Images of tumors derived from mice treated with drugs. (B) H&E images of the major organs. Scale bar, 50 nm. The regions circled with the black lines indicate the location of the potential lesion. (C) Volume of MCF-7 ADR tumors isolated from mice. (D) Relative volume of MCF-7 ADR tumors isolated from mice. (E) Weight of MCF-7 ADR tumors isolated from mice. (F) Relative body weight of MCF-7 ADR tumor-bearing mice. Data are shown as the mean ± SD, *n* = 5, **p < .01, ***p < .001, N.S. indicates *p >* .05.

Chemotherapeutic agents are often unable to effectively access the tumor region due to unstable circulating drug concentrations and insufficient tumor targeting, both of which will inevitably lead to systemic side toxicity, especially in drug-resistant patients (Xu et al., 2016). Nanoscale drug delivery vehicles are considered to possess the ability to ‘target’ cancer regions *via* the EPR effect (Zhaorigetu et al., 2003). From an *in vivo* analysis of fluorescent images (supporting materials, Figure S8), sericin-PBLG-DOX micelles were found to be able to access the cancer region within 24 h, indicating the EPR effect of these micelles. To examine the *in vivo* anti-tumor efficacy of sericin-PBLG-DOX against ADR, we utilized MCF-7 ADR tumor-bearing nude mice (HepG2 ADR cells were unable to form solid tumors according to the ATCC literature). Upon the tumor volume reaching 200 mm^3^, the mice were administered four intravenous injections (3 d apart) of saline, sericin-PBLG, free DOX (5 mg/kg), or sericin-PBLG-DOX (5 mg/kg DOX). Compared with the saline group, the sericin-PBLG-DOX group showed a suppression of tumor growth by almost 70%, which was dramatically higher than the approximately 30% inhibition achieved by free DOX (Figure 6(A,C)). Both the relative tumor volume and tumor weight showed the same tendencies (Figure 6(D,E)), indicating that the sericin-PBLG-DOX delivery system could effectively reverse drug resistance *in vivo*. Previous studies have shown that at the final endpoint studies, the tumor volumes in sericin-PBLG-treated animals were slightly lower than those in saline-treated animals, indicating that sericin might exhibit chemotherapeutic effects by suppressing tumorigenesis in animal models (Zhaorigetu et al., 2003; ZhaorigetuYanaka et al., 2003; Zhaorigetu et al., 2007). However, in this study, the relative tumor volumes between these two groups showed no significant difference. Consequently, this anti-tumor effect might be because of difference in the primary tumor volumes before drug treatment, since the tumor volume in the saline-treated group was higher than in the sericin group.

To further evaluate the potential toxic side effects of sericin-PBLG-DOX, we monitored changes in body weight throughout the experiment. Weight loss was only observed in the free DOX group, and there were no obvious weight changes observed in the other three groups (Figure 6(F)). A histological analysis of the main organs (heart, liver, spleen, lungs, kidneys, and brain) using H&E staining further confirmed the safety of the sericin-PBLG-DOX treatment, as there were no pathological changes compared with the saline and sericin-PBLG groups (Figure 6(B)). However, some changes (the lesion regions circled by black lines), including heart myocardial (fibril loss and neutrophil infiltration) and liver damage (congestion and morphological changes), were observed in the free DOX group (Gabizon et al., 1986). All of these data confirm the superiority of the sericin-PBLG-DOX micelles over free DOX. Taken together, these results provide strong evidence that sericin-PBLG-DOX not only effectively overcomes cancer drug resistance but also induces negligible systemic toxicity, suggesting that sericin-PBLG-DOX micelles have potential value for clinical translation.

## 4. Conclusion

We fabricated novel DOX-loaded sericin micelles, with enhanced cellular uptake and a pH-triggered intracellular drug release, which could effectively reverse drug resistance.

Sericin-PBLG micelles exhibit a high drug loading capacity with a high stability, which contributes to a prolonged blood circulation time. These micelles also had an appropriate size distribution, a negative surface potential, and good biocompatibility. The nanoparticles could be efficiently internalized into cells through clathrin-mediated endocytosis. Sericin-PBLG-DOX was transported to, and accumulated within, perinuclear lysosomes that were physically distant from the transmembrane drug pumps, subsequently releasing DOX under the low pH microenvironment. The released DOX directly entered the nucleus, leading to DNA damage and an enhanced anti-tumor effect. The underlying mechanism, by which sericin-PBLG-DOX traffics to the lysosomes and activates the caspase-dependent apoptosis pathway, requires further investigation.

As a result of the design on these nanocarriers, the systemic administration of sericin-PBLG-DOX micelles can achieve a sufficiently high local drug concentration that enhances the chemotherapeutic effect of DOX toward tumors, thus reducing undesired systemic cytotoxic side effects, including damage to the heart, liver, kidneys, lungs, brain, and spleen. Sericin-PBLG-DOX micelles are an effective and safe system for the delivery of chemotherapeutic drugs and provide a potential method to reverse multi-drug resistance.

The diversity and abundance of sericin’s side chains also allow for further modification to meet clinical requirements, which could provide ample opportunities to meet the demand for broad functional versatility in personalized and precision cancer medicine.

## Supplementary Material

Supporting_files___4-23_.docx
