# Supplementary material for: Sericin nanomicelles with enhanced cellular uptake and pH-triggered release of doxorubicin reverse cancer drug resistance
Source: Drug Deliv. 2018 May 9;25(1):1103–16. doi: 10.1080/10717544.2018.1469686 (PMC6058513; doi:10.1080/10717544.2018.1469686)
Supplement: Supporting_files___4-23_.docx [file IDRD_A_1469686_SM3196.docx]

**Supporting files**

**Methods:**

1. ***Preparation of sericin powder***

Sericin powder was prepared as reported previously with modification. Briefly, silkworm cocoons (*Bombyx mori*) were boiled in Na_2_CO_3_ solution（0.02 M）for 0.5 hour and then the supernatant solution was filtrated to remove the insoluble. Subsequently, this solution was dialyzed to against deionized water using cellulose dialysis membranes (molecular weight cutoff ranging from 8 to 14 kDa) for 3 days to remove salt and centrifuged with 10000 rpm for 10 min to remove aggregates. Finally, Sericin powder was obtained through lyophilization and stored in 4℃ under dry condition.

1. ***Sodium dodecyl sulfate polyacrylamide gel electrophoresis（SDS-PAGE）assay***

About 0.1-0.2 mg of the sericin samples were loaded onto a 8% sodium dodecyl sulfate (SDS) polyacrylamide gel electrophoresis (PAGE) and separated by electrophoresis. After that, the gel was stained with PAGE silver staining kit (Solarbio, China). And imaged using Gel Doc EZ (Bio-Rad, USA).

1. ***In vitro immunotoxicity assay***

The mouse macrophage (RAW264.7) cells were seeded into CLSM dishes at the density of 1×10^5^ for 24h. After cells were cultured at 37 °C for 24 h, the media were replaced with fresh media containing sericin-PBLG (400ug/ml), or lipopolysaccharide(1ug/ml) (as a positive control). After a 24 h incubation, cells were washed with PBS three times, fixed with 4% paraformaldehyde for 10 min at room temperature, washed with PBS for another three times, and then treated with 1% bovine serum albumin (BSA) for 30 min. After the removal of the BSA solution, 0.1% triton X-100 was added for a 5-min incubation. After washed with PBS for three times, the cells were stained with rhodamine-phalloidin (Abcam, USA) for 60 min in dark and then with 4′, 6-diamidino-2-phenylindole（DAPI) for 15 min. After PBS wash, the stained cells were imaged using CLSM (Olympus, Japan).

1. ***The construction of DOX resistance cells: MCF-7 ADR cells and HepG2 ADR cells.***

MCF-7 and HepG2 cells were exposed to doxorubicin (DOX) at an initial concentration of 0.025 mg/L. Subsequently, the survival cells which were tolerant to DOX, were selected for further cultured in a double concentration of doxorubicin. This procedure was repeatedly applied to the cells, until the cells could finally be cultured well in the presence of 1.0 mg/L doxorubicin. When the cells were totally tolerance to doxorubicin, the ADR cells were successfully established. The ADR cells were continuously exposed to certain doxorubicin concentrations for resistance maintenance.

1. ***DNA damage assay***

MCF-7/ADR cells and HepG2 ADR cells were seeded into CLSM dishes at the density of 1×10^5^ for 24h. Cells treated with 2ml DOX or sericin-PBLG-DOX solutions (the concentration of DOX was 16ug/ml). After incubation for 48 hours, the cells were washed and ﬁxed by 4% paraformaldehyde for 10 min. Subsequently, the cells were stained with 1% Trition-100 for 30min, 5% BSA for 30min, anti-γH2AX (Abcam) antibodies for 12 hours, fluorescent secondary (Abcam) antibody, and finally 4',6-diamidino-2-phenylindole (DAPI) for cell nuclei imaging. The dishes were imaged using CLSM (Olympus, Japan).

1. ***In vivo distribution study***

As a step towards developing a tumor model, MCF-7 ADR cells were harvested at density of 1 × 10^8^/mL, suspended in PBS, after which 50 µL of the suspension was injected into the right flank of the mice. Upon the tumor volume exceeding 200 mm^3^, sericin-PBLG-DOX (5 mg/kg DOX) was applied via tail vein injection. The strongly fluorescent dye, Dir, which is triggered by near-infrared light, was also encapsulated in the sericin-PBLG-DOX micelles. The mice were imaged using a small animal *in vivo* fluorescence imaging system (IVIS® Lumina III, PerkinElmer, USA), and their major organs were collected for fluorescence imaging.

1. ***Quantitative reverse transcription PCR (qRT-PCR) analysis***

Total RNA from cultured cells was extracted with RNAiso Plus reagent (Takara, China).500ng total RNA was reversely transcribed into cDNA by Prime Script RT Master Mix (Takara) according to the manufacturer’s protocol. Relative mRNA level was assessed by qPCR with SYBR Premix Ex Taq kit (Takara) and normalized to GAPDH with the following primers: P-gp forward (F), 5′-GCTGGGAAGATCGCTACTGA-3′ and 5′-GGTACCTGCAAACTCTGA-3′. GAPDH F, 5`-CAGGAGGCATTGCTGATGAT-3` and R, 5`-GAAGGCTGGGGCTCATTT-3`. Changes in the expression were calculated by the 2-ΔΔCT method.

1. ***Western-blot test analysis:***

MCF-7 ADR cells were incubated with DOX or sericin-PBLG-DOX (the concentration of DOX was 16ug/ml) for 48 hours. Then total protein was extracted from treated cell samples. Then the samples were treated with two different methods:

8.1 ProteinSimple capillary electrophoresis immunoassay (ProteinSimple, Santa Clare, CA, U.S.A.) was performed according to the ProteinSimple user manual. In brief, whole-cell extract samples (800 ng per lane) were mixed with a master mix (ProteinSimple) to a final concentration of 1*9 sample buffer, 1*9 fluorescent molecular weight markers, and 40 mmol L^-1^ DTT and then heated at 95 °C for 5 min. The samples, blocking reagent, primary antibodies including P-gp (CST, USA), Caspapse-3 (CST, USA) and Cleaved Caspase-3 (CST, USA), horseradish peroxidase-conjugated secondary antibodies, chemiluminescent substrate and separation and stacking matrices were also dispensed to a designated wells plate. The electrophoresis and immunodetection steps took place in the capillary system and were fully automated using instrument default settings. The digital image was analyzed and quantified with Compass software (ProteinSimple) after normalization using GAPDH (loading control).

8.2 Equal volumes of protein were loaded onto a 10% sodium dodecyl sulfate polyacrylamide gel and separated by electrophoresis. The resulting protein bands were transferred to polyvinylidene difluoride membranes (Bio-Rad). The membranes were blocked for 1 hour in tris-buffered saline (TBST) buffer containing 5% non-fat milk. Immunoblotting was performed by incubating the membranes in 5% milk-TBST at 4°C overnight with primary antibodies against P-gp (1:1000, CST). The membranes were washed three times in TBST and incubated with a secondary antibody horseradish peroxidase-conjugated goat anti-rabbit IgG (1:1000, CST) for 1 hour. Then, the membranes were again washed three times in TBST, and the antibodies were detected by enhanced chemiluminescence (Millipore). Densitometric analysis using Quantity One software (Bio-Rad) was used for quantification, and the results were normalized to GAPDH.

**Results and discussion**

1. ***The molecular weight of sericin***

The molecular weight of sericin has been detected by sodium dodecyl sulfate polyacrylamide gel electrophoresis（SDS-PAGE）assay (Fig. S1), indicating that the molecular weight of sericin ranges from 15 to 70kDa.


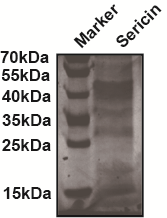


*Figure S1: 8% SDS-PAGE analysis of sericin from cocoons.*

1. ***The graft ratio of PBLG (GR) in sericin-PBLG.***

Table S1 Element analysis results of Sericin and Sericin-PBLG

| Sample | C/% | H/% | N/% |
| --- | --- | --- | --- |
| Sericin-PBLG | 42.5 | 6.1 | 13.7 |
| Sericin | 41.4 | 6.4 | 15.2 |

N ratio in BLG monomer was 10.6%, the graft ratio of PBLG (GR) in Sericin-PBLG can be calculated by N’s variation. GR(%)=(N_seicin_-N_sericin-PBLG_)/(N_seicin_-N_BLG monomer_) = (15.18-13.7)/(15.18-10.6) =32.3%. The details have been added in the revised supporting data (Table.S1).

PBLG was grafted onto sericin by ring-opening polymerization (ROP) of (γ-benzyl-L-glutamate N-carboxyanhydeide (NCA), triggered by primary amine groups (-NH2) on sericin backbone, the number of primary amine groups involved in ROP reaction was uncertain, so the degree of polymerization of PBLG couldn’t be calculated.

1. ***In vitro immunotoxicity of sericin-PBLG nanomicelles***

The immunotoxicity of sericin-PBLG was examined using murine macrophage-like cells (RAW264.7) that respond to inflammatory agents, such as lipopolysaccharides (LPS), by increasing plasma membrane spreading. Compared to LPS treatment that activated the cells, sericin-PBLG treatment did not cause cell spreading (Fig. S2), instead morphologically similar to the untreated control, indicating that sericin-PBLG does not elicit inflammatory responses at the cellular level. This data furtherly demonstrates that sericin-PBLG possess a good biocompatibility


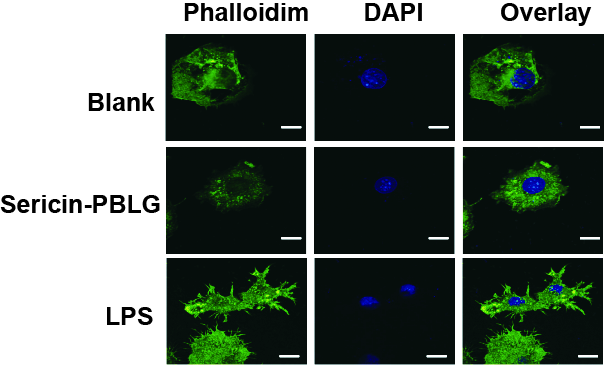


Fig. S2. The confocal images showing the morphology of RAW264.7 cells before (control) and after the treatments with LPS and sericin-PBLG for 24 h. Rhodamine-phalloidin (red) and DAPI (blue) are used to stain F-actin and nuclei, respectively. Scale bar, 20 μm

1. ***TEM images of ADR cells after incubation with sericin-PBLG-DOX***

Dozens of vesicles containing black particles, were observed inside the cell cytoplasm (Fig. S3). We hypothesized the black particles might be the sericin-PBLG-DOX nanomicelles, and these nanomicelles were absorbed through endocytosis pathway.


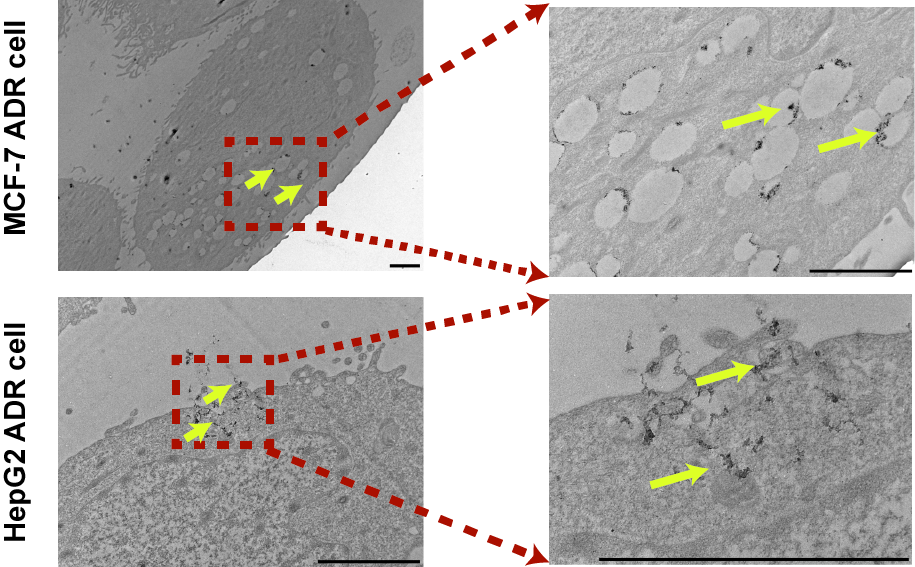


Fig. S3. TEM images of endocytosis by MCF-7 ADR and HepG2 ADR cells, yellow arrows represent sericin-PBLG-DOX nanomicelles. Scale bar, 2 µm.

1. ***Viabilities of MCF-7/S, HepG2/S, MCF-7 ADR and HepG2/ADR cells after incubation with DOX***

Chemotherapeutic resistance whether it is inherent or acquired has become the greatest challenge to effective systemic therapy in majority of cancers. The aim of the construction of sericin-PBLG-DOX is to reverse drug resistance. In order to address issues, we firstly constructed the DOX-resistance cells including MCF-7 ADR and HepG2 ADR cells. In MCF-7 ADR cells, the half-maximal inhibitory concentrations (IC_50_) of free DOX was dramatically higher than those of MCF-7 /S cells (149.28μg/mL), as the IC_50_ of free-DOX (149.28μg/mL) was 86.8-fold higher than that of drug sensitive cells (1.72μg/mL) (Fig. S4A). Consistently, IC_50_ of free-DOX (116.4μg/mL) in HepG2 ADR cells, was 70.1-fold higher than that of HepG2/S cells (1.66μg/mL) (Fig. S4B). This data shows that free-DOX is less sensitive in ADR cells, indicating ADR cells are successfully constructed.

**
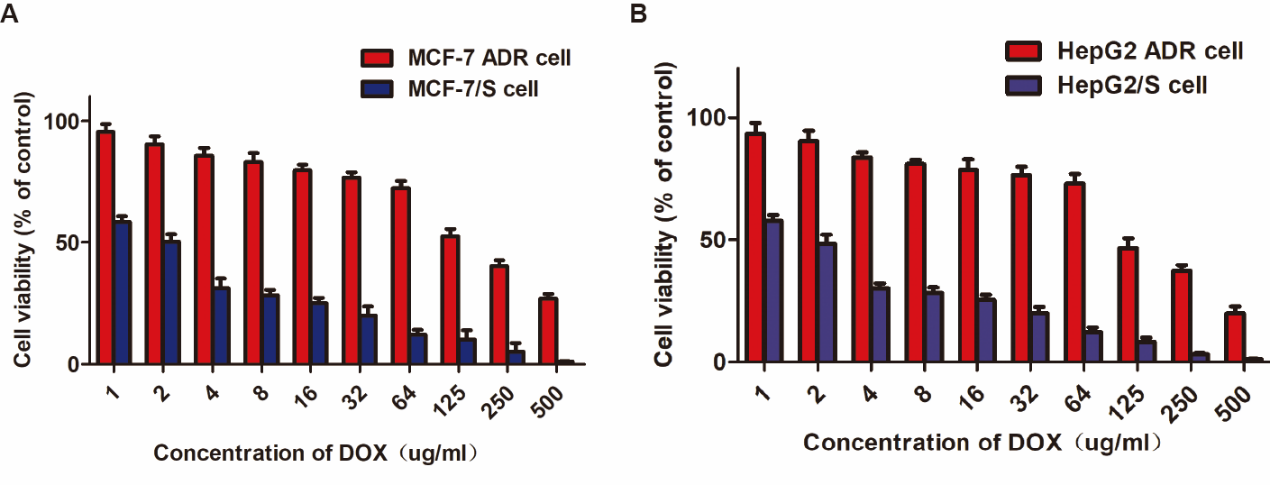
**

Fig. S4. (A) Viabilities of MCF-7 ADR and MCF-7/S cells after incubation with DOX at different concentration. (B) Viabilities of HepG2 ADR and HepG2/S cells after incubation with DOX at different concentration.

1. ***DNA damage assay***

Nanomedicines are supposed to increase the DOX accumulation inside drug resistance cancer cells, thus induce more DNA damage, as the DOX could inhibit the synthesis of DNA and RNA. Since that, for the enhancing cellular uptake of nanomicelle, the sericin-PBLG-DOX might achieve more DOX accumulation inside the cancer cells, then induce more DNA damage. To examine that, we detected the expression of anti-gamma H2A.X (γH2AX). The fluorescence of the DNA damage sensor γH2AX was dramatically elevated in the sericin-PBLG-DOX group, as the other three groups showed no obvious difference. H2AX is a classic DNA damage marker, as DNA damage occurs, the H2AX protein could be phosphorylate, and transferred into γH2AX. Consequently, higher expression of γH2AX was observed in sericin-PBLG-DOX group (Fig. S5), indicating that sericin-PBLG-DOX can effectively reverse drug resistant.
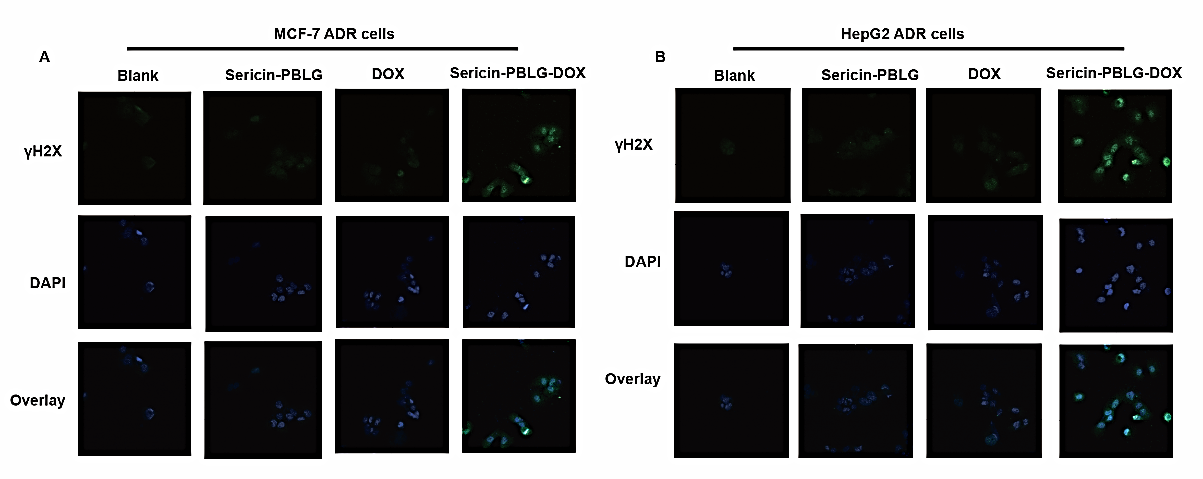


Fig. S5. (A) Expression of γH2AX of MCF-7 ADR cells after incubation with sericin-PBLG, DOX and sericin-PBLG-DOX for 48 hours. (B) Expression of γH2AX of HepG2 ADR cells after incubation with sericin-PBLG, DOX and sericin-PBLG-DOX for 48 hours.

1. ***The expression of P-gp protein in MCF-7 ADR and HepG2/ADR cells after incubating with sericin-PBLG-DOX***

The enhanced activity and high expression of the drug efflux transporter P-glycoprotein (P-gp), which locates on the tumor cell membrane, is critical for drug resistance. The transmembrane structure allows P-gp to actively pump out endocytosed drugs, subsequently reducing the intracellular drug accumulation and causing reduced chemotherapeutic efficacy. The expression of P-gp is supposed to be upregulated in drug resistance cell. In our study, the expression of P-gp was much higher in ADR cell at mRNA level, compared to drug sensitive cells (Fig. S6A). Meanwhile, its protein level showed the same tendency (Fig. S6B). Consequently, the overexpression of P-gp, as a characteristics of drug resistant cell, was furtherly proved in the ADR cells we constructed.


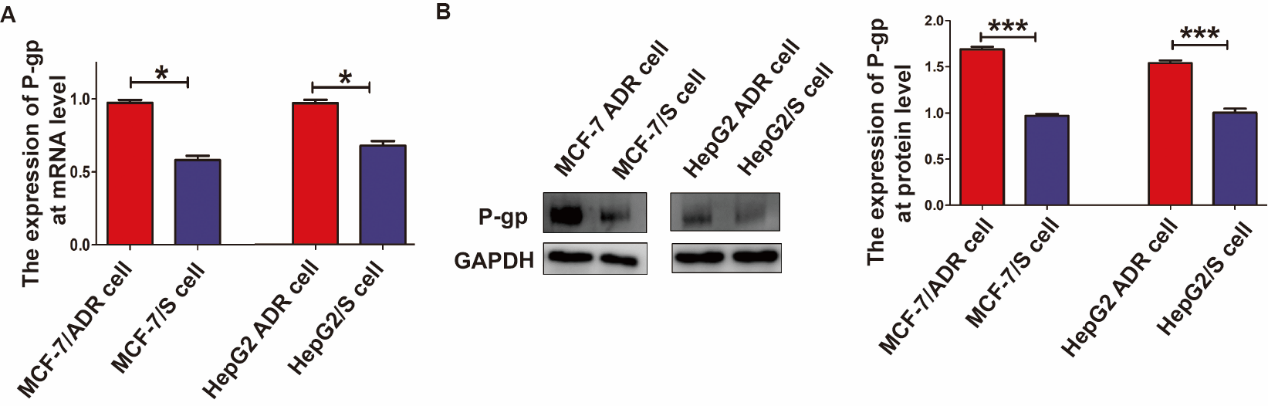


Fig. S6. (A) Expression of P-gp protein at mRNA level in MCF-7 ADR, MCF-7/S, HepG2 ADR and HepG2/S cells. (B) Expression of P-gp protein at protein level in MCF-7 ADR, MCF-7/S, HepG2 ADR and HepG2/S cells. * indicates *P*＜0.05, *** indicates *P*＜0.001.

1. ***The expression of P-gp protein in MCF-7 ADR and HepG2/ADR cells after incubation with sericin-PBLG-DOX***

Disrupting the function of P-gp by competitively inhibiting the binding sites of P-gp, or reducing the expression of P-gp, are promising means of reversing drug resistance. In our study, the enhanced anti-tumor effect of sericin-PBLG-DOX was observed. However, whether the function of P-gp was inhibited or not, remains unknown. From the results (Fig. S7A and B), neither of the presence of DOX, sericin-PBLG or sericin-PBLG-DOX, yielded any changes in P-gp expression. Consequently, these results indicate that sericin-PBLG-DOX, but not free DOX, can effectively eliminate drug-resistant cancer cells by enhancing intracellular delivery, instead of inhibiting P-gp.

**
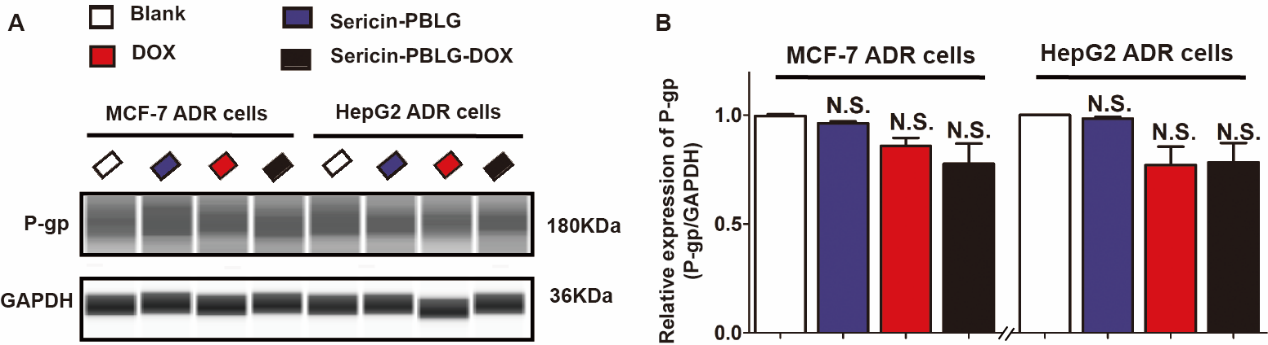
**

Fig. S7 (A) Expression of P-gp protein in MCF-7 ADR cells after incubation with sericin-PBLG, DOX and sericin-PBLG-DOX for 48 hours. (B) The corresponding quantification of gray value of P-gp protein. N.S. indicates no significant difference.

1. ***In vivo biodistribution study***

Nanoscale drug delivery vehicles have been considered to possess the ability to “target” cancer regions via the EPR effect. To address this issue, we first constructed nude mice bearing MCF-7 ADR tumors (HepG2 ADR cells were unable to form solid tumors). Then 100 µL of diR iodide (1,1‘-dioctadecyl-3,3,3’,3‘-tetramethylindotricarbocyanine iodide) labeled nanomicelles called DIR-sericin-PBLG-DOX, was administered to these tumor-bearing mice via a tail vein injection. Fluorescence images (Fig. S8A) were captured after 24 hours using a carestream imaging system. After 24 hours, the treated rats were sacrificed with a pentobarbital overdose, and the tumors were excised and imaged using a small animal in vivo fluorescence imaging system (Fig. S8B). The *in vivo* fluorescence images indicate that the sericin-PBLG-DOX nanomicelles could access the cancer region in 24 hours Consistently, the immunofluorescence imaging revealed large levels of drug accumulation in tumor region (Fig. S8C). Due to the high uptake activity of liver macrophages and fluorescent instability of free DIR in the body, free DIR mainly accumulates in liver and spleen, without specific high tumor-targeting ability. These results indicated that DIR-labelled sericin micelles showed well cancer-targeting ability, which could not be observed in free DIR group. These data further validate the stability and EPR effect of these micelles.


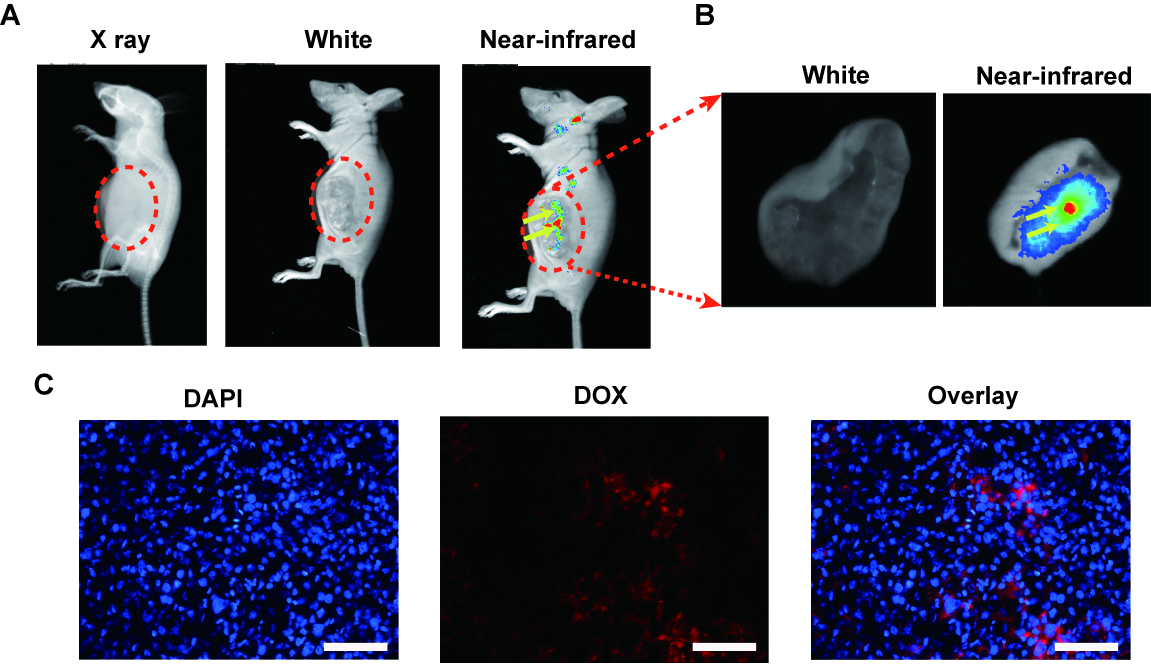


Fig. S8. In vivo bio-distribution study. (A) Fluorescence images of mice at 24 hours after treatment with sericin-PBLG-DOX. (B) Fluorescence image of tumor. (C) Immunofluorescence images of tumor. Scale bar, 500 nm. The red circles mean the tumor, the yellow arrow means the nanomicelle.
